# Early-life behavior, survival, and maternal personality in a wild marsupial

**DOI:** 10.1093/beheco/arad070

**Published:** 2023-09-14

**Authors:** Weliton Menário Costa, Wendy J King, Timothée Bonnet, Marco Festa-Bianchet, Loeske E B Kruuk

**Affiliations:** Division of Ecology & Evolution, Research School of Biology, Australian National University, Canberra Australian Capital Territory, 2601, Australia; Division of Ecology & Evolution, Research School of Biology, Australian National University, Canberra Australian Capital Territory, 2601, Australia; Département de biologie, Université de Sherbrooke, 2500 Boulevard de l’Université, Sherbrooke, Québec, J1K 2R1, Canada; Division of Ecology & Evolution, Research School of Biology, Australian National University, Canberra Australian Capital Territory, 2601, Australia; French National Centre for Scientific Research, Centre d’Études Biologiques de Chizé, UMR 7372 CNRS-La Rochelle Université, 79360 Villiers en Bois, France; Division of Ecology & Evolution, Research School of Biology, Australian National University, Canberra Australian Capital Territory, 2601, Australia; Département de biologie, Université de Sherbrooke, 2500 Boulevard de l’Université, Sherbrooke, Québec, J1K 2R1, Canada; Division of Ecology & Evolution, Research School of Biology, Australian National University, Canberra Australian Capital Territory, 2601, Australia; Institute of Ecology and Evolution, School of Biological Sciences, University of Edinburgh, Edinburgh EH9 3FL, UK

**Keywords:** animal personality, early-life behavior and survival, macropods, maternal variance, multivariate Bayesian statistics

## Abstract

Individual behavior varies for many reasons, but how early in life are such differences apparent, and are they under selection? We investigated variation in early-life behavior in a wild eastern gray kangaroo (*Macropus giganteus*) population, and quantified associations of behavior with early survival. Behavior of young was measured while still in the pouch and as subadults, and survival to weaning was monitored. We found consistent variation between offspring of different mothers in levels of activity at the pouch stage, in flight initiation distance (FID) as subadults, and in subadult survival, indicating similarity between siblings. There was no evidence of covariance between the measures of behavior at the pouch young versus subadult stages, nor of covariance of the early-life behavioral traits with subadult survival. However, there was a strong covariance between FIDs of mothers and those of their offspring tested at different times. Further, of the total repeatability of subadult FID (51.5%), more than half could be attributed to differences between offspring of different mothers. Our results indicate that 1) behavioral variation is apparent at a very early stage of development (still in the pouch in the case of this marsupial); 2) between-mother differences can explain much of the repeatability (or “personality”) of juvenile behavior; and 3) mothers and offspring exhibit similar behavioral responses to stimuli. However, 4) we found no evidence of selection via covariance between early-life or maternal behavioral traits and juvenile survival in this wild marsupial.

## Teaser

We report evidence for consistent individual differences in behavior among eastern gray kangaroos. Behavioral variation was apparent at a very early stage of development (still in the pouch in the case of this marsupial), and between-mother differences could explain personality variation in juvenile behavior. In addition, mothers and offspring showed similar behavioral responses to stimuli. However, there was relationship between early-life or maternal behavioral traits and juvenile survival.

## INTRODUCTION

Behavior varies both within and between individuals. Interest in consistent behavioral differences between individuals has generated the field of animal “personality,” and with it substantial research on the causes and consequences of individual behavioral variation (Laskowski et al. 2022). These differences may be family-specific: for any trait, behavioral, or otherwise, offspring of different mothers (referred to here as “non-siblings”) are likely to differ in phenotype due both to differences in the genes they have inherited, and to maternal effects, which may be genetic or environmental (Mousseau and Fox 1998; Räsänen and Kruuk 2007). For behavioral traits, differences between offspring of different mothers may contribute to repeatable individual variation and, hence, to measures of individual personality. The extent of this maternal-level variation can be estimated using the same variance-component approach used to estimate individual repeatability of behavioral traits from between-individual variance in behavior (Dingemanse and Dochtermann 2013). If heritable genetic or maternal effects are important sources of individual differences (Taylor et al. 2012; White and Wilson 2019; Zablocki-Thomas et al. 2019), they will play an important role in determining offspring behavioral repeatability. In mammals, maternal effects are especially important for early-life traits but typically decline as offspring age (e.g. Wilson and Réale 2006; Gauzere et al. 2020), thus maternal effects on offspring behavior may play an especially important role early in offspring life. Understanding the causes of variation in behavior is particularly relevant when behavioral differences have fitness consequences (Smith and Blumstein 2008). If variation in behavior is associated with differences in survival or reproduction, it is expected to be shaped by natural selection (Travis and Reznick 2018). Maternal effects on offspring behavior could, therefore, have adaptive implications (Wolf and Wade 2001).

Individual differences in maternal behavior can also affect offspring fitness. For example, maternal protection from conspecific attacks and duration of mother–offspring association are correlated with offspring size and mass in chimpanzees (*Pan troglodytes*; Samuni et al. 2020). Maternal behavior is also associated with offspring growth and fitness in spotted hyenas (*Crocuta crocuta*; Höner et al. 2010), gray seals (*Halichoerus grypus*; Hall et al. 2001), North American red squirrels (*Tamiasciurus hudsonicus*; Westrick et al. 2020), chimpanzees (*P. troglodytes*; Murray et al. 2018; Schülke et al. 2019), and eastern gray kangaroos (*Macropus giganteus*; King et al. 2017). Maternal and offspring behavioral traits are correlated in several mammals, including personality traits in North American red squirrels (Taylor et al. 2012) and gray mouse lemurs (*Microcebus murinus;*Zablocki-Thomas et al. 2019), and dominance rank in rhesus macaques (*Macaca mulatta*), baboons (*Papio* sp.), vervet monkeys (*Cercopithecus aethiops sabaeus*) (see review: Maestripieri 2018), and spotted hyenas (East et al. 2009). These correlations may be partly driven by genetic effects, and there is an increasing evidence of heritability of behavioral traits across a range of species (Weiss et al. 2000; Dingemanse et al. 2002; Williamson et al. 2003; Taylor et al. 2012; Dochtermann et al. 2015; Zablocki-Thomas et al. 2019). To determine a trait’s heritability, the similarity between parents and offspring in a trait, measured as the slope of regression of offspring on parent values, can provide an upper limit of heritability within a population, though the estimate may be inflated by “common environment” effects (Kruuk and Hadfield 2007; Thomson et al. 2018). However, estimation of these parameters in wild populations can be challenging in natural conditions as it requires identification and monitoring of parents and offspring and their behaviors (Archard and Braithwaite 2010). As a result, most studies of the heritability of behavior have used either captive-bred individuals or wild animals tested in artificial environments (Dochtermann et al. 2019) (but see Saastamoinen et al. 2018, on dispersal).

In this study, we investigated variance and covariance in juvenile behavior and survival in a wild population of eastern gray kangaroos. We also quantified how these traits varied between offspring of different mothers and how they covaried with maternal behavior. Studies of marsupials can offer unparalleled insights into early juvenile development because of the possibility of measuring phenotypes of very young offspring while still in the pouch, a stage that is comparable to late gestation in eutherian mammals. Our eastern gray kangaroo study population in Victoria, Australia, has been the subject of long-term monitoring (Montana et al. 2020). Previous work in the population has shown that maternal body size and condition are positively correlated with offspring survival, growth, and maternal fecundity (Quesnel et al. 2017, 2018; Mackay et al. 2018). In another marsupial, the brushtail possum (*Trichosurus vulpecula*), maternal mass is correlated with offspring age at sexual maturity, and with dispersal of sons (Bannister et al. 2019). Here, we extend the study of maternal effects in marsupials. We provide, to our knowledge, the first analysis of the behavior of pouch young (PY) in the wild, of maternal variation in behavior, and of the heritability of behavioral traits in marsupials. We analyzed two measures of offspring behavior (activity as PY and flight initiation distance (FID) as subadult) and one measure of adult behavior of mothers (flight initation distance), and then investigated possible associations of all of these measures with survival of the offspring. Using the rare cases of adoption in the population (King et al. 2015), we also compared the behavior of biological versus adoptive mothers and their young, which could give insights into the contribution of heritable genetic versus maternal shared-environment effects to any mother–offspring correlations in behavior. We thus addressed four questions:

1) Do maternal siblings behave similarly to each other, and differently from non-siblings, in early life?
2) Is either offspring early-life behavior or maternal behavior associated with offspring survival to weaning?

Further, focusing on the trait of FID in subadults, we asked:

3) What is the contribution of variation between offspring of different mothers to the repeatability of offspring behavior (or “personality”)?
4) Are maternal and offspring behaviors correlated, and do these associations differ for biological versus adoptive mothers?

## METHODS

### Study population

We studied a wild population of marked eastern gray kangaroos at Wilsons Promontory National Park, VIC, Australia (38°57ʹS, 146°17ʹE). In this population, adult females usually reproduce annually, with a maximum of one offspring per year. Offspring stay in the pouch for approximately 10 months, and then continue to be nursed until approximately 19 months of age (Poole et al. 1982; King and Goldizen 2016). Most offspring are born from November to May; for this analysis, cohorts are named after the calendar year for January of a given breeding season (e.g. an offspring born in either December 2017 or January 2018 would both be assigned to the 2018 cohort). Survival rates of juveniles vary widely across years (range 8–89%), possibly due to variation both in the density of likely predators, in particular foxes (*Vulpes vulpes*) and in weather conditions (Plaisir et al. 2022; Bergeron et al. 2023). Approximately 75–85% of kangaroos in the study area were individually marked and monitored for survival and reproduction each year, with a resighting probability of 99% for females and 92% for males (Bergeron et al. 2023). Captured individuals were ear-tagged with a unique color combination at first capture; this includes any PY weighing >900 g (King et al. 2011). We considered individuals aged 3 years and older as adults.

This study involved individuals born in four cohorts (2016–2019) and their mothers, with behavioral observations and PY measures made in 2017, 2018, and 2019. In these years, there were 276–336 marked individuals in the study population, comprising 115–140 adult females, about 55 adult males, 50–85 subadults, and 30–55 PY. We estimated the birth dates of PY based on body size measurements (Poole et al. 1982). We classified the developmental stage of young as: small PY (unfurred, small distension of pouch, aged ≤ 3 months); medium PY (not completely furred, head sometimes out of medium-sized pouch, aged 4–6 months); large PY (completely furred, often with head outside pouch, aged 7 months or older); and 1-year-old offspring (out of the pouch but unweaned) (Jaremovic and Croft 1991).

Mothers were typically captured from August to November, when PY were aged about 7–9 months and were making only occasional exits from the pouch (Poole 1975). Mothers were sedated at capture, but their PY were not (King et al. 2011). While we collected measurements of the mother, her young remained inside the pouch. We then extracted the young from the pouch for measurement and tagging, and while we weighed the mother. We returned the young to the pouch after an average of 8 min. As described below, we scored the behavior of each young during its short removal from the pouch. We also did not measure behavior of the mother within a few days following capture.

### Definitions of traits

We recorded data on four traits: 1) movement of PY during handling at capture; 2) FID, of subadults aged 13–35 months; 3) FID of adult females; and 4) survival of subadults to weaning at 21 months. We describe these in turn below; summary statistics and sample sizes for each trait are given in Table 1.

**Table 1.** Means, standard deviations, and sample sizes for all traits.

Trait	Number of observations	Number of individuals	Mean # of observations per individual	Number of mothers	Mean # of young per mother	Trait mean
Pouch Young (PY) Movement (score 0–1)	126	126	1	87	1.4 (±0.6)	0.47 (±0.24)
Subadult Flight Initiation Distance (meters)	490	107	4.6 (±2.2)	86	1.2 (±0.5)	6.6 (±4.4)
Adult Female Flight Initiation Distance (meters)	875	156	5.6 (±3.2)	–	–	6.6 (±3.7)
Subadult Survival (binary)	181	181	1	111	1.6 (±0.8)	0.67 (±0.47)

Pouch Young Movement is a score from 0 to 1 (see “Methods” section); FID is in meters (m); Subadults consists of individuals aged 13-35 months. Subadult Survival scores survival from 6 to 21 months (0 died, 1 survived). Subadult Survival data includes but is not limited to individuals in the PY Movement and Subadult FID datasets. The mean difference in age between siblings of the same mother was 1.7 (±0.7) years (range 1–3 years).

1) PY movement

We assessed PY behavior by scoring body movements across eight stages of handling at the point of capture of the mother: extraction from the mother’s pouch, placement of the offspring inside a cloth bag, weighing, collection of three body measurements (foot, hind leg, and head length), tagging, and being held briefly inside an observer’s jacket (for warmth) while the mother was weighed. We recorded 1/0 for any presence/absence of movement at each stage, summed these values across all points to a maximum of 8, then divided by 8 to estimate an individual score ranging from 0 to 1. For practicality, in 2019, we combined responses for foot and hind-leg length measurements and for extraction from the pouch and placement inside a cloth bag, to give a total of only six stages of handling, but again deriving a 0-1 score. The mean total duration of the handling process across all years was 7.9 (±1.9 SD) minutes. The PY data (PY movement) were assessed for the 2017, 2018, and 2019 cohorts, at 6.4–10.8 months of age. The average score of PY Movement was 0.47 (±0.24 SD, range 0–1). All analyses of PY Movement included age at capture (mean 8.0 ± 0.8 SD months) as a covariate in the model, to account for any changes in behaviour with age.

2) Subadult FID and (3) Adult Female FID

We measured FID of subadult offspring (age range 13–35 months) and of adult females, as the distance to the nearest metre at which a kangaroo moved away when approached by a human (Strong et al. 2017). High values of FID indicated a response to the approaching human at a greater distance, whereas low values indicated that the individual allowed the human to come closer before it moved, implying a “bolder” response. The observer started at 30 m from a target kangaroo that was positioned alone or in the periphery of a group, walked directly toward that individual at a constant pace, and stopped when the individual took flight. The terrain was flat and there were no obstructions that could limit the view of the approach. We tested one individual per group per approach, in group sizes of 1 to 6. In groups, the target individual was always the closest to the observer. We always tested mothers and their offspring on different dates, and we also always avoided tests when they were within 3 m of each other. FID measures were made in 2017, 2018, and 2019. Juveniles from the 2016 and 2017 cohorts thus had their responses measured at ages 1 and 2 years, whereas juveniles from the 2018 cohort were measured at age 1 year only. The 2019 cohort was not tested for FID. We measured the FID of 156 adult females between 2017 and 2019; of these 156 females, we also had measures of PY Movement or Subadult FID measures of 87 (Table 1).

For all measures of FID, we also noted the date, time, geographic position, group size, and the presence of the mother or an offspring in the same group when applicable. Geographic positions were recorded with a Garmin^®^ GPSMAP 64 device, as an east–west coordinate in meters. To determine membership of groups, we followed a “10-m chain rule” (Jarman 1987; King et al. 2015), so individuals were considered to be in the same group if they were within 10 m of at least one other group member. “Group size” was the number of individuals aged 1+ years in a group. For each individual being tested for FID, we also classified the presence of its mother or an offspring within the group as a factor: alone, that is, neither a mother nor an offspring present; mother present (for 1-year-olds and 2-year-olds); small PY present (for adult females); medium PY present (for adult females); large PY present (for adult females); and 1-year-old offspring (for adult females). To avoid an extra parameter in the models, the rare cases of a female with both a PY and a yearling were considered within the class of their PY if carrying a large PY, but within the class of 1-year-old offspring if carrying a small or medium-stage PY. Subadults were tested with their mother in the same group in 31.4% of tests, but never at the same time. For adult females, 20.9% of the tests were with a small PY, 6.6% with a medium PY, 18.7% with a large PY, 9.4% were with a 1-year-old in the same group, and 44.3% were without an offspring in the same group.

Finally, to account for any habituation via multiple testing of FIDs, we recorded the test number and the number of days since the previous test for that individual and included these variables in our models. Test number indicated whether it was the first, second, or *n*th time an individual was being tested for FID. The number of days since the previous test accounted for the variation in time between trials that resulted from repeats both within and between years. As this could not be defined for the first test, the first value was set to an arbitrary value larger than the largest possible number of days since the previous test (500 days; use of a different value did not affect the results).

FID measures were available on more adult females than just the mothers of the offspring considered in this analysis. We, therefore, analyzed all available data to provide the best estimates of variation in adult female FID. Ninety-nine percent of subadult FID trials were carried out by one observer, WMC, however 9% of FID trials on adult females were carried out by another trained observer, so we included observer ID as a two-level factor in the model of adult female data.

4) Subadult survival

We monitored subadult survival from 6 to 21 months for each individual, by which age nearly all young are weaned and have passed their second winter (King et al. 2017). Survival was measured for all four cohorts (2016–2019).

See Table 1 for sample sizes and summary statistics for all traits.

### Ethical note

Population monitoring and captures were undertaken with ethics approval from the Université de Sherbrooke (permit no.s MFB2016-01, MFB2020-01), the University of Melbourne (no. AEC 1312902.1), and the Australian National University (no. A2018/02) and research permits from the Victorian Department of Environment, Land, Water & Planning (nos.10007062, 10008630). Behavioral experiments and observations were conducted with animal ethics approval from the Australian National University (nos. A2017/17, A2018/02).

### Statistical analysis

We fitted two multivariate generalized linear mixed models using a Bayesian Monte Carlo Markov Chain framework in the statistical package *MCMCglmm* (Hadfield 2010). Code for the MCMCglmm models is provided in the Supplementary Material. We used Model I to address question 1 (do siblings behave similarly to each other?) and question 2 (are either offspring early-life behavior or maternal behavior associated with offspring survival?). We used Model II for question 3 (what is the contribution of variation between mothers to the repeatability of offspring behavior?) and question 4 (are maternal and offspring behaviors correlated?).

Model I was a four-trait GLMM with response variables of PY Movement, Subadult FID, Adult Female FID (including mothers), and Subadult Survival. Although we had repeated measures of Subadult FID, to facilitate fitting of this complex four-trait model, we averaged the multiple measures on each offspring into a single mean value. Each offspring was, therefore, represented by a single observation for each trait. However, each mother could be represented by multiple offspring and also by multiple observations for her FID (average 5.6 FID observations per individual, range 1–14; Table 1).

For all four traits, we fitted Mother ID as a random effect to estimate between-mother variances (or “maternal repeatability”) and covariances for offspring traits and individual-level repeatability for Adult Female FID. Maternal repeatability measures the level of consistent differences between non-siblings’ behavior and indicates similarities among siblings: variance between mothers indicates covariance within mothers. For the offspring traits (PY Movement, Subadult FID, and Subadult Survival), the residual variance in the model represents within-mother between-offspring variance or differences between siblings. We acknowledge that using mean Subadult FID may lead to some overestimation of maternal repeatability of Subadult FID in this model and that an explicit model of repeated measures with error would have been preferable (see Ponzi et al. 2020, Dingemanse et al. 2021), but repeated measures of Subadult FID are considered in more detail in Model II below.

For PY Movement, we fitted fixed effects of age at capture (in months), sex, and year (a 3-level factor: 2017, 2018, and 2019). For Subadult FID and Subadult Survival, we fitted fixed effects of sex and year (year was a 3-level factor for FID and a 4-level factor for Subadult Survival). For Adult Female FID, we also included fixed effects of GPS location along an east–west axis to capture the spatial variation in the study area (Menário Costa 2021), group size, presence of an offspring of different stages (as defined above), test number, number of days since the previous test, year, and a two-level observer effect.

The MCMCglmm runs generated posterior distributions of parameter estimates, from which we could estimate posterior means (for fixed effect parameters), posterior modes (for variance–covariance parameters and ratios), and 95% highest posterior density credible intervals (CIs) for each parameter and also for any derived estimates. For each offspring trait, we estimated *maternal repeatability* by dividing the estimate of the variance between mothers by the total variance in the offspring traits (defined as the sum of all the variance components). For Adult Female FID, we estimated *individual repeatability* by dividing the between-individual variance by the total variance. Calculations were made on each sample of the posterior distribution to generate posterior distributions of the two repeatability estimates, from which we estimated posterior modes and 95% CIs. As Subadult Survival was a binary trait (0/1), we fitted it specifying the family “categorical” in MCMCglmm. In a binomial model, residual effects have a variance fixed at 1, but can still covary with other random effects. Because parameter estimates from the Subadult Survival model were on the latent (logit) scale, we used the QGicc function in the package QGglmm (de Villemereuil et al. 2016; de Villemereuil 2018) to back-transform the latent-scale variance estimates and also to calculate repeatability on the original data scale as well as on the latent scale (see equations below).

We estimated the latent-scale repeatability in Subadult Survival as:


$$\begin{array}{llll} & Maternal\;\;repeatability\; & =\frac{var\_Mother}{var\_Mother+var\_Residual},\;\; & \;\;\;where\;var\_Residual=1 \end{array}$$ (1)

The data-scale repeatability was estimated as:


$$\begin{array}{lll} & Maternal\;repeatability\; & =\frac{var\_Mother}{var\_Mother+var\_Residual+var\_Binomial\;sampling} \end{array}$$ (2)

where “var_Residual” =1, and “var_Binomial sampling” is the variance related to binomial sampling (Nakagawa and Schielzeth 2010; de Villemereuil et al. 2016).

We used Model I (the four-trait model) to estimate the covariances between the four traits. In particular, we estimated maternal-level covariance between the Mother ID effects, for all behavioral responses, to test for maternal-level associations across offspring traits (e.g. do mothers whose offspring have high PY Movement also have offspring with high FID?), and for associations between maternal FID and offspring average behavior and Subadult Survival (e.g. do mothers with a high FID have offspring with high PY Movement, and do they have offspring with high survival rates?). We also fitted residual covariances for offspring traits to test for within-mother–offspring-level associations (e.g. for an individual offspring is high PY Movement associated with high FID?). For the random effect Mother ID, a covariance matrix between traits was set using an unstructured covariance structure in MCMCglmm (Hadfield 2017). We also fitted the covariance for the residual variance–covariance of the three offspring traits. We could not estimate residual covariance with the Adult Female FID because this was the only trait measured on adults, so we fixed this covariance to zero.

Model II was a bivariate model for Subadult FID and Adult Female FID, and was used to estimate the contribution of between-mother variation to the repeatability of juvenile behavior, and also the covariance between mother and offspring FID. This model included multiple measures on each offspring for Subadult FID (average 4.6 observations per individual, range 1–10; Table 1). For the response variable Subadult FID, we fitted random effects of Mother ID and Offspring ID, and for the response variable Adult Female FID, we fitted a random effect of Mother ID. For both traits, we fitted the fixed effects of GPS location along the east–west axis, group size, presence of the mother or offspring, test number, the number of days from the previous test, and year. Further, for Subadult FID, we fitted fixed effects of age at observation (in months, as a covariate) and sex, and for Adult Female FID, a two-level effect of observer.

As in Model I, we fitted the covariance between the Mother ID effects across the behavioral responses. We fixed the covariance to zero for the residual effects, as there could be no covariance across the two traits in within-individual effects.

Both Models I and II were run for 1300 × 10^3^ iterations, with a burn-in of 300 × 10^3^, a thinning interval of 1 × 10^3^ and an inverse-Wishart prior distribution. We report the posterior distribution mode and 95% CIs for each parameter, considering there to be statistical support for the covariance of random effects if the 95% CIs did not overlap 0 and, for fixed effects, if *p*MCMC (Probability of Markov Chain Monte Carlo) was <0.05.

For Subadult FID in Model II, we estimated *maternal repeatability* by dividing the variance between mothers (Mother ID variance) by the total variance, and *individual repeatability* by dividing the variance between offspring (Offspring ID variance) by the total variance as above. For Adult Female FID, we estimated *individual repeatability* by dividing the variance between individuals (Mother ID variance) by the total variance. Model II only had Gaussian-family traits, so unlike Model I, we did not need to convert repeatability to the data scale in Model II.

In the Subadult FID response, we then estimated *total repeatable* variance between individuals from the variance between mothers plus the additional variance between offspring, and hence a *total repeatability* by dividing this sum by the total variance:


$$\begin{array}{lll} & Total\;repeatability\;in\;subadult\;FID\;\; & =\frac{var\_Mother+var\_Of\;\;fspring}{var\_Mother+var\_Of\;\;fspring+var\_Residual}. \end{array}$$ (3)

Finally, we estimated the proportion of total repeatability (offspring plus maternal variance, as in a model without Mother ID the between-mother variance would be attributed to between-offspring variance) that could be ascribed to maternal-level effects as:


$$\begin{array}{lll} & Maternal\;proportion\;of\;total\;repeatability\; & =\frac{var\;\_\;Mother}{var\;\_\;Mother+var\;\_\;Of\;fspring}. \end{array}$$ (4)

### Mother-offspring regression for FID

We did not have a sufficiently informative multi-generational pedigree to estimate heritability of FID from a quantitative genetic “animal model” (Kruuk 2004). Instead, we used the estimates of the phenotypic covariance between mothers’ and offspring’s FID to calculate the mother–offspring regression slope, and hence (having doubled the estimate; Falconer and Mackay 1996) to provide an upper limit on the heritability $upper\;\_\;h^{2}$ (Falconer and Mackay 1996), noting that this is very likely to be inflated by maternal or shared environmental effects (Lande and Price 1989; Kruuk and Hadfield 2007):


$$\begin{array}{l} upper\_h^{2}=2\;\times \frac{cov(Mother,\;Of\;fspring)}{var\_Mother}. \end{array}$$ (5)

Note that both Models I and II provided estimates of the covariance between mothers and offspring in FID and the variance between mothers, and hence an estimate of the regression slope. We opted to use the estimates from Model II only to calculate $upper\;\_\;h^{2}$, as it was a more detailed FID model, using the repeated measures for both mothers and offspring. We estimated the $upper\;\_\;h^{2}$, as described above, for each sample of the posterior distribution, and report the resulting posterior mode and 95% CI.

Finally, we made use of the (rare) occurrence of adoption in kangaroos (King et al. 2015) and examined the association between mothers and offspring in six cases of adoption in 2016 and 2017: 4 involving reciprocal switches and 2 involving mothers whose biological young disappeared. All adoptions occurred when PYs were aged 8–10 months. The adoptive mother was responsible for all subsequent maternal care until weaning, about 6–9 months later (King et al. 2015). We repeated the analysis of the association between Adult Female FID and Subadult FID using observations on adopted individuals for which we had measured FID for the young and both the biological and adoptive mothers. Despite the very small sample size, this analysis could potentially indicate the relative importance of genetic and non-genetic causes of similarity in behavior.

## RESULTS

**Figure 1.**
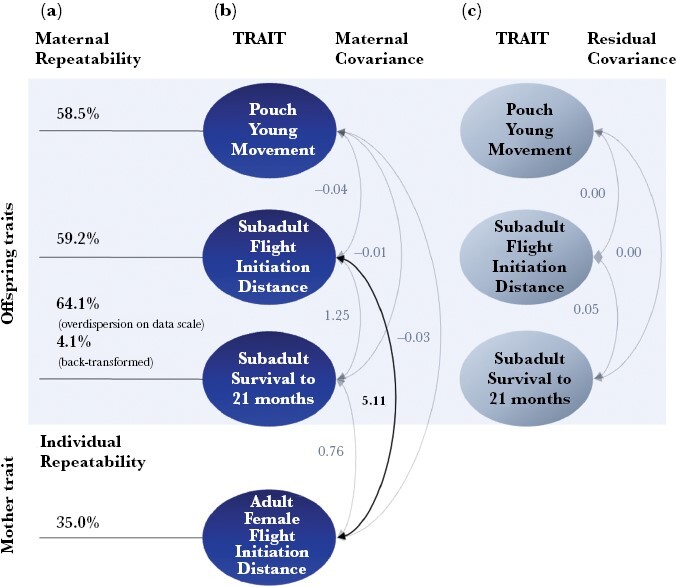
Mother and offspring (co)variances estimated from Model I (Table 2) for eastern gray kangaroos. (a) *maternal repeatability* estimates for offspring traits, and *individual repeatability* for Adult Female FID (Table 2c), (b) maternal-level covariances across offspring and mother traits (Table 2b), (c) residual (offspring-within-mother) covariances across offspring traits (Table 2b). Bold indicates statistical support for non-zero estimates of the parameter. Arrows link pairs of traits for which covariance estimates are shown.

**Figure 2.**
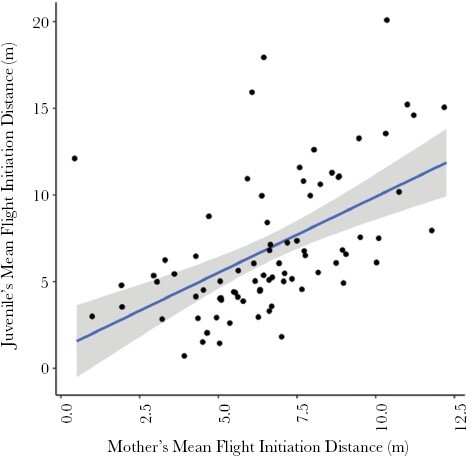
Relationship between mother and subadult offspring mean FIDs (*n* = 86 mothers) in eastern gray kangaroos. The lines indicate the linear regression, and the shading the 95% confidence intervals. The “upper limit heritability” values derived from these data were greater than 1, suggesting that maternal environmental effects inflated the covariance (see the main text).

We present below the results from the two models addressing our four questions. The main results for Model I (the four-trait model) are in Table 2 and Figure 1, and the main results for Model II, focusing on FID, are in Table 3 and Figure 2; further details for both models are available in Supplementary Tables S1 and S2.

**Table 2.** Summary of the four-trait Model I, analyzing repeatability in the behavior of eastern gray kangaroos, with response variables

	PY Movement		Subadult FID		Subadult Survival		Adult Female FID	
(a) Fixed effects	Estimate	*p-MCMC*	Estimate	*p-MCMC*	Estimate	*p-MCMC*	Estimate	*p-MCMC*
Intercept	0.16 (−0.62, 0.88)	0.662	**5.56 (4.47, 6.67)**	**<0.001**	**1.02 (0.08, 1.85)**	**0.022**	−1.10 (−8.56, 7.65)	0.778
Sex (Female)	0.09 (−0.04, 0.22)	0.200	0.50 (−0.87, 1.74)	0.418	−0.41 (−1.30, 0.43)	0.360	**–**	**–**
Year (base 2017)								
–2018	−0.15 (−0.29, 0.02)	0.076	**1.58 (0.28, 3.08)**	**0.026**	−0.40 (−1.42, 0.86)	0.478	0.10 (−0.64, 0.85)	0.746
–2019	0.08 (−0.05, 0.23)	0.240	**2.51 (1.14, 3.99)**	**0.004**	**2.62 (0.56, 4.79)**	**0.004**	0.98 (−0.43, 2.52)	0.164
–2020	**–**	**–**	**–**	**–**	0.28 (**–**0.67, 1.38)	0.592	**–**	**–**
(b) Random effects	Variance–covariance matrices							
	PY movement		Subadult FID		Subadult **S**urvival		Adult female FID	
Mother ID								
PY Movement	**0.10 (0.07, 0.14)**							
Subadult FID	−0.04 (−0.34, 0.23)		**7.89 (2.90, 13.54)**					
Subadult Survival	−0.01 (−0.13, 0.11)		1.25 (−0.84, 3.38)		**1.54 (0.30, 3.33)**			
Adult Female FID	−0.03 (−0.25, 0.21)		**5.11 (2.40, 7.66)**		0.76 (−0.58, 2.06)		**4.94 (3.37, 6.67)**	
Residual								
PY Movement	**0.08 (0.05, 0.10)**							
Subadult FID	0.00 (−0.23, 0.23)		**7.56 (4.57, 11.61)**					
Subadult Survival	0.00 (−0.08, 0.09)		0.05 (−2.16, 2.25)		Fixed to 1			
Adult Female FID	–		–		–		**8.54 (7.74, 9.47)**	
(c) Variance proportions								
	PY Movement		Subadult FID	Subadult Survival (latent scale)	Subadult Survival (data scale)		Adult Female FID	
Maternal repeatability	**58.5% (46.7%, 68.1%)**		**59.2% (26.4%, 75.7%)**	**64.1% (34.3%, 82.9%)**	**4.1% (1.6%, 11.4%)**		**35.0 (28.5%, 45.2%)**	
Residual (latent scale = overdispersion on data scale)	**–**		**–**	**36.0% (17.1%, 65.7%)**	**3.8% (2.7%, 5.8%)**		**–**	
Residual (data scale)	**41.5% (31.9%, 53.3%)**		**40.8% (24.3%, 73.6%)**	-	**92.1% (88.1%, 98.8%)**		**65.1% (54.8%, 71.5%)**	

PY Movement (*n* = 126), Subadult FID (one average value per individual, *n* = 107), Subadult Survival (*n* = 181), and Adult Female FID (*n* = 156). We show only the fixed effects of sex and year as they were more important for our questions in this study; see Supplementary Table S1 for estimates of all variables included in the model. (a) fixed effects of sex and year for each response variable. The “base” level for sex is male, and for year is 2017. (b) random effects: (co)variance estimates from posterior distribution mode and respective 95% CI in parentheses: variances are on the diagonal and covariances below diagonal. (c) Estimates of individual and maternal repeatability (posterior mode) followed by the 95% CI in parentheses. “–” indicates that the variable was not fitted in the model. Bold indicates *p*MCMC (Markov Chain Monte Carlo) <0.05 for fixed effects or that the CIs were separated from zero for random effects and repeatability estimates. In (c), the “maternal repeatability” means “individual repeatability” for the Adult Female FID traits (see main text), and we included data on all adult females, not only mothers of subadults in the sample.

**Table 3.** Random effect results from the bivariate Model II, using repeated measures of FID for each juvenile (*n* = 107) and adult female kangaroo (*n* = 156).

	Subadult FID	Adult female FID
(a) Random effects (variance and covariance estimates)		
Offspring ID variance	3.61 (1.55, 6.37)	–
Mother ID: variance–covariance matrix		
Trait: Subadult FID	4.97 (1.45, 8.63)	
Trait: Adult Female FID	4.14 (1.98, 6.11)	4.78 (3.07, 6.44)
Residual variance	8.11 (7.08, 9.45)	8.50 (7.70, 9.45)
(b) Variance proportions		
Maternal repeatability	27.5% (12.4%, 45.7%)	–
Individual repeatability	21.6% (9.3%, 37.1%)	38.9% (27.5%, 45.1%)
Residual	48.5% (37.7%, 60.3%)	61.1% (54.9%, 72.5%)
Total repeatability	51.5% (39.7%, 62.3%)	
Maternal proportion of total repeatability	55.3% (26.6%, 80.4%)	–

Fixed effect parameters are in Supplementary Table S2. (a) Random effect variance–covariance estimates. (b) Proportions of total variance contributed by maternal, individual, and residual variances. Total repeatability is defined as the sum of between-mother (maternal repeatability) and between-individual (individual repeatability) proportions. Estimates are followed by the 95% CIs in parenthesis.

1) Do siblings behave similarly to each other and differently from non-siblings?

We found support for maternal repeatability in both PY Movement and Subadult FID (Table 2b, Supplementary Table S1, and Figure 1). PY Movement and Subadult FID had maternal repeatability of 58.5% and 59.2%, respectively (Table 2c), with CIs clearly separated from zero, so the majority of variance in these offspring traits could be ascribed to different mothers. For Subadult Survival, maternal repeatability accounted for a large part of the total variation on the latent scale (64.1%, Table 2c). When we back-transformed the GLMM latent-scale estimates for Subadult Survival to proportions on the data-scale, adding the stochastic variation associated with moving from the expected to observed data scale (Nakagawa and Schielzeth 2010; de Villemereuil et al. 2016), the overall maternal repeatability estimates decreased to 4.1%, but with CIs still separated from zero (Table 2c). The back-transformation thus illustrates the dramatic change in estimates of proportions depending on whether estimates are considered on latent versus data scales.

2) Is either offspring early-life behavior or maternal behavior associated with offspring survival to weaning?

Considering the covariances estimated in Model I, there was no support for any association of either offspring or maternal behavioral traits with Subadult Survival. This was the case both across mothers (Table 2b, “Mother ID” covariance), in that there was no indication that groups of siblings with higher average levels of PY Movement or Subadult FID had higher (or lower) average Subadult Survival, nor within mothers (Table 2b, “Residual” covariance), as there were no associations at the level of individual offspring.

From the estimates of the other covariances in Table 2b, there was also no support for any association between offspring behavior at the different life stages, namely between PY Movement and Subadult FID (Table 2b). The covariance between Subadult FID and Maternal FID was large, however, and we consider this association in more detail with Model II below.

3) Do maternal effects contribute to the repeatability of offspring behavior?

Model II focused on the repeated measures of FID in both offspring and mothers (Table 3). Maternal repeatability was 27.5% for Subadult FID in this model (Table 3b), lower than the value estimated in Model I, possibly due to the averaging within offspring for Model I. A further 21.6% of the variance was due to differences between individuals (“individual repeatability” over and above maternal repeatability, Table 3b), so the “total repeatability” of Subadult FID was 51.5%. Variation between mothers, therefore, accounted for 55.3% (95% CI: 26.6%, 80.4%) of the total repeatability of Subadult FID. The individual repeatability of Adult Female FID was 38.9% (Table 3b).

4) Are maternal and offspring behaviors correlated, and do these associations differ for biological vs adoptive mothers?

We found a strong association between measures of FID of mothers and their offspring (Figure 2; FIDs were always estimated at different times, and mothers and their offspring were together in only a minority of tests). Models I and II both estimated positive covariances between maternal and offspring FID, though the estimate was slightly higher in Model I (5.11 (95% CI: 2.40, 7.66), Table 2b) versus 4.14 (95% CI: 1.98, 6.11), Table 3a). The upper limit of heritability, upper_h^2^, estimated from Model II (Equation (5)) was 1.73 (95% CI: 1.42, 1.95). The estimate was greater than 1, which should not occur for narrow-sense genetic heritability (Falconer and Mackay 1996); as we discuss below, this indicates that shared common environmental effects inflated the covariance between mothers and offspring.

The supplementary regressions on the FIDs on the very small sample size of adopted young (Supplementary Table S3, *n* = 6) returned equivalent upper-limit heritability estimates of 0.75 (95% CI: −6.22, 21.02) for the biological mother and 1.26 (95% CI: −16.07, 22.41) for the adoptive mother. Although the adoptive mother estimate was nearly twice that of the biological mother, the values had very large uncertainty reflecting the small sample sizes, so it was not possible to determine the relative impact of direct genetic effects and maternal environmental effects.

## DISCUSSION

Our analyses indicate differences between offspring of different mothers (non-siblings) in early-life behavior and survival. There was no indication of covariance between juvenile or maternal behavior and subadult survival. Mothers and offspring, however, had similar FIDs. Below, we discuss the implications of these results for our understanding of individual behavioral differences.

We found evidence of “maternal repeatability,” or differences between offspring of different mothers, in all three offspring traits, including PY movement, subadult FID, and subadult survival, indicative of genetic or maternal effects on these traits. Maternal repeatability estimates the part of total variation in offspring behavior explained by juveniles having different mothers, or a measure of the level of non-sibling differences, and hence sibling similarities, in behavior. It, therefore, incorporates both strict-sense “maternal effects” and genetic effects, where the term “maternal effect” refers to the impact a mother has on her offspring over and above the direct effects of the genes they inherit from her. These maternal effects can in turn be driven by both environmental or genetic differences between mothers (Rossiter 1996; Reinhold 2002; Räsänen and Kruuk 2007). Maternal effects during early development may be especially important in mammals, as offspring depend on maternal provisioning through gestation and lactation (Reinhold 2002). Mothers may thus be expected to have the most influence on offspring phenotype at these early stages before other external influences increase in relevance (East et al. 2009; Maestripieri 2018; Samuni et al. 2020). These effects also include influences on early-life offspring survival (Nafus et al. 2015; Quesnel et al. 2017; Westrick et al. 2020). In the broader sense, maternal effects can be quantified from the differences between offspring of different mothers, but such differences will also include effects of genetic variance if that variance is not estimated separately (Kruuk and Hadfield 2007). As estimated here, we have not attempted to separate maternal effects from genetic effects, which will also generate similarities between offspring of the same mother, but such analyses will be feasible with ongoing work to estimate relatedness from genomic data for the kangaroo study population.

Marsupials offer an especially interesting opportunity to investigate phenotypic variation at very early developmental stages, but to our knowledge no study has considered the components of variance in behavior in wild marsupial PY. Some studies have addressed the influence of maternal traits on marsupial offspring survival in the brushtail possum (Bannister et al. 2019) and, in our study population, in eastern gray kangaroos (Quesnel et al. 2017, 2018), but none have explicitly quantified maternal repeatability. Our results here show support for maternal repeatability in both behavior and survival of offspring. Variance between mothers in these offspring traits suggests genetically and/or environmentally induced effects on juvenile offspring phenotype in a wild social marsupial. Evidence of maternal repeatability in PY movement is particularly interesting because it reveals differences between offspring from different mothers at a very early life stage comparable to a fetus in late gestation in eutherian mammals (Tyndale-Biscoe and Renfree 1987).

In subadult survival, we found support for maternal repeatability, which accounted for a large part of the total variance (on the latent-scale estimates), suggesting that there is a large variance in fitness among females relative to other modeled terms. However, there was no evidence of covariance with the maternal behavioral trait. From a life-history evolution perspective, offspring survival is sometimes treated as a component of maternal total fitness (Wolf and Wade 2001), but the absence of any covariance between adult female FID and offspring survival suggests that maternal FID is not under selection via offspring survival in this population. In contrast, other studies in different species and investigating different behavioral traits have found evidence for selection of maternal behavior in relation to offspring survival. For example, in North American red squirrels, maternal aggressiveness is correlated with offspring survival (Boon et al. 2007), and there are genetic and maternal effect correlations between activity and aggression (Taylor et al. 2012). Maternal dominance is correlated with offspring survival to 24 months in spotted hyenas (Hofer and East 2003; Höner et al. 2010) and with stress hormone levels in baboon subadult males (Onyango et al. 2008). Maternal sociality is linked with offspring mortality via infanticide in white-faced capuchin monkeys (*Cebus capucinus*; Kalbitzer et al. 2017).

In subadult FID, we found that 55% of the total repeatability was explained by differences in the mothers of the juveniles (maternal repeatability) compared to less than half being explained by differences between juveniles themselves (individual repeatability). To our knowledge, only a few studies to date have tried to isolate such effects during repeatability estimation (Taylor et al. 2012; White and Wilson 2019; Zablocki-Thomas et al. 2019). This separation is important because genetically and/or environmentally induced effects can contribute to differences between individuals. Some researchers recommend not to “correct” for intrinsic or extrinsic sources of individual heterogeneity when analyzing personality, as these may be relevant to the individual differences observed in the population (Wilson 2018). However, it is still valid to ask how much of the total repeatability is actually due to maternal influences, in the same way as analyses may consider how much of repeatability is due to sex, age, or environmental effects (Bell et al. 2009).

Furthermore, in subadult FID, we found support for covariance with mothers’ FID (arguably a measure of personality; Figure 2). Despite the rapid expansion of the animal personality field (Beekman and Jordan 2017), the investigation of how maternal personality contributes to offspring trait variation has received less attention (Reddon 2012; Schuett et al. 2013). In mammals, most investigations into heritability of personality have been restricted to primates and ungulates (Dochtermann et al. 2019). Our analyses estimate an upper limit on the heritability of FID based on the regression of offspring values on maternal values. We only had information on the resemblance between mothers and offspring, though ideally, we would have fitted a model estimating both additive genetic variance and maternal effects variance separately, to separate the shared-genes effects from maternal environmental effects (Kruuk and Hadfield 2007). Some studies of personality using this approach have found support for heritability of behavioral traits (Taylor et al. 2012; Dochtermann et al. 2015; Zablocki-Thomas et al. 2019). Given the definition of heritability as the proportion of total phenotypic variance due to additive genetic variance (Falconer and Mackay 1996), the value of heritability should lie between 0 and 1. Our results show an unrealistically large upper-limit heritability estimate (substantially >1), presumably because environmental effects inflated similarities between mothers and offspring. Our regression has also not been age-standardized, with the maternal and offspring phenotypes being measured at different ages, which may affect the upper-limit estimates (Chevin 2015). A study on zebra finches (*Taeniopygia guttata*) cross-fostered offspring and found that “personality” variation arose primarily as a maternal effect derived from the foster mother, indicating that shared environmental effects may be more important than genes in shaping personality variation in that system (Schuett et al. 2013). For the few cases of adoption in our population, the covariance of offspring with the foster mother was nearly twice as large as that with the biological mother, indicating a similar trend to that found in zebra finches, but high uncertainty precluded meaningful conclusions. If we ignore the uncertainty due to small sample sizes and consider the biological mother association as the better estimate of heritability, the upper-limit heritability estimate is around 70% for FID; however, this estimate may still be confounded by environmental and maternal effects prior to the adoption. Future analyses with genomic data for the study population will allow us to separate the different environmental and genetic factors contributing to phenotypic variance in eastern gray kangaroos.

Several aspects of the environment could cause mothers and offspring to have similar personalities. Mother and offspring share physical and social environments, particularly when the juvenile is 10–18 months old, as they are closely associated before weaning (King and Goldizen 2016). As we did not find any correlation between mother’s FID and offspring behavior when the young were in the pouch (PY movement, measured at ~8 months old), it is possible that the offspring learn a level of responsiveness to threats from their mother during the first days after pouch exit (~10-month-old), a critical life stage (Breed and Sanchez 2010). After that stage, the offspring behavior does not seem to change with age or conditions. We always tested mother and offspring FID responses at different times and in most cases when they were not grouped together. Subadult FID did not change with age (across the period 1–2 years old, changing from dependent to independent of maternal care) and the presence of the mother in the group did not affect the subadult FID (Supplementary Table S2). In the minority of cases when they were in the same group, both did not always take flight, and when both did, sometimes the young fled first, other times the mother did. Therefore, the similarities in FID are likely not the result of the offspring copying their mother’s immediate behavior during the test (or vice versa), but possibly suggest that offspring FIDs are learned early in life and/or inherited. A laboratory study on a lizard, *Liopholis whitii* (Munch et al. 2018), housed offspring either with their mother or alone during the first 8 weeks of life and found that maternal presence affected juvenile expression of three key behaviors (latency in time until offspring emerged after a threat, time spent moving in the familiar environment, and time spent moving in a novel environment) compared to individuals housed alone. The lizard study did not quantify the similarities in behavior between mother and offspring in either treatment but provided evidence that even relatively simple forms of mother–offspring association early in life can significantly impact offspring behavioral phenotypes.

In conclusion, our study revealed that variation in behavior and early survival of juveniles is influenced by differences between offspring of different mothers and that these maternal differences also explained a large part of individual differences in the personalities of a wild marsupial. Juvenile behavior covaried with maternal behavior, with the similarity potentially driven by both genetic and shared environmental effects. Mothers are an important source of variation in behavior and survival of juveniles, and more studies using multivariate approaches will contribute to our understanding of the causes and consequences of variance and covariance between individuals in their behavior at different stages of life.

## Supplementary Material

arad070_suppl_Supplementary_Material

## Data Availability

Analyses reported in this article can be reproduced using the data provided by Menário Costa et al. (2023). Statistical code is provided in the Supplementary Material.

## Notes

We acknowledge the intellectual, technical, logistic, or financial support of partners: fieldwork funding from Natural Sciences and Engineering Research CouncilDiscovery Grants to MFB, Holsworth Wildlife Research Endowment & Ecological Society of Australia (2017–2019, Round 1) funding to WMC, and an Australian Research Council Laureate Fellowship to LEBK; scholarship funding to WMC from the Australian National University; field assistance from Parks Victoria, Carolina Dolan, Emily Hynes, Luca Montana, Marc-Antoine Poirier, Charles-Alexandre Plaisir, Rebecca Phillips, Martin Taube, Lynden Costin, and others who conducted long-term monitoring previously; logistic assistance from Parks Victoria, University of Melbourne, and the Australian National University; manuscript comments from Kristal Cain, Elissa Cameron, Celine Frère, and three anonymous reviewers; and general discussions and advice from Rob Magrath, Naomi Langmore, and other colleagues in the Division of Ecology and Evolution and the Biological Data Science Institute at the Australian National University.
