# Supplementary material for: Early-life behavior, survival, and maternal personality in a wild marsupial
Source: Behav Ecol. 2023 Sep 14;34(6):1002–12. doi: 10.1093/beheco/arad070 (PMC10636729; doi:10.1093/beheco/arad070)
Supplement: arad070_suppl_Supplementary_Material [file arad070_suppl_supplementary_material.docx]

# Supplementary Materials

Contents

[Supplementary Materials 1](#_Toc58362273)

[Table S1. Results from Model I: multivariate with a random effect of mother. 2](#_Toc58362274)

[Table S2. Results from Model II: bivariate with random effect of offspring and mother. 4](#_Toc58362275)

[Table S3. Results from the supplementary test on adoptive young FID 6](#_Toc58362276)

[MCMC glmm Codes for Model I 7](#_Toc58362277)

[MCMC glmm Codes for Model II 11](#_Toc58362278)

Table S1. Complete fixed effect results from the four-trait Model I, analyzing repeatability in behavior of eastern grey kangaroos, with response variables: Pouch Young (PY) Movement, Subadult Flight Initiation Distance (FID, averaged), Juvenile Survival, and Maternal FID. The ‘base’ levels for categorical variables were: *sex* male, *presence of offspring* none, *year* 2017, and *observer* Observer 1. Bold indicates *p*MCMC (Markov Chain Monte Carlo) < 0.05. Iterations = 750001:3247501. Thinning interval = 2500. Sample size = 1000. DIC: 5213.001.

|  | PY Movement | | Subadult FID | | Juvenile Survival | | Maternal FID | |
| --- | --- | --- | --- | --- | --- | --- | --- | --- |
| Fixed effects | Estimate | *p-MCMC* | Estimate | *p-MCMC* | Estimate | *p-MCMC* | Estimate | *p-MCMC* |
| Intercept | 0.16 (-0.62, 0.88) | 0.662 | **5.56 (4.47, 6.67)** | **<0.001** | **1.02 (0.08, 1.85)** | **0.022** | -1.10 (-8.56, 7.65) | 0.778 |
| Age at Capture (months) | 0.03 (-0.06, 0.13) | 0.500 | - |  | - |  | - |  |
| Sex (Female) | 0.09 (-0.04, 0.22) | 0.200 | 0.50 (-0.87, 1.74) | 0.418 | -0.41 (-1.30, 0.43) | 0.360 | - |  |
| East-west Coordinate (in meters) | - |  | - |  | - |  | 0.001 (0.000, 0.002) | 0.058 |
| Group Size | - |  | - |  | - |  | **-0.24 (-0.49, -0.02)** | **0.050** |
| Presence of offspring: |  |  |  |  |  |  |  |  |
| - mother with Small Pouch Young (<3 months-old) | - |  | - |  | - |  | -0.50 (-1.19, 0.24) | 0.168 |
| - mother with Medium Pouch Young (4-6 month) | - |  | - |  | - |  | 0.87 (-0.04, 1.83) | 0.076 |
| - mother with Large Pouch Young (7+ month) | - |  | - |  | - |  | **1.29 (0.66, 1.93)** | **<0.001** |
| - mother with 1-year-old Offspring | - |  | - |  | - |  | 0.74 (-0.06, 1.51) | 0.070 |
| Test Number | - |  | - |  | - |  | -0.14 (-0.31, 0.05) | 0.140 |
| Difference in Days from the Previous Test | - |  | - |  | - |  | 0.001 (0.000, 0.003) | 0.076 |
| Year (relative to 2017) |  |  |  |  |  |  |  |  |
| -2018 | -0.15 (-0.29, 0.02) | 0.076 | **1.58 (0.28, 3.08)** | **0.026** | -0.40 (-1.42, 0.86) | 0.478 | 0.10 (-0.64, 0.85) | 0.746 |
| -2019 | 0.08 (-0.05, 0.23) | 0.240 | **2.51 (1.14, 3.99)** | **0.004** | **2.62 (0.56, 4.79)** | **0.004** | 0.98 (-0.43, 2.52) | 0.164 |
| -2020 | - |  | - |  | 0.28 (-0.67, 1.38) | 0.592 | - | - |
| Observer (Observer 2) | - |  | - |  | - |  | 0.63 (-0.44, 1.79) | 0.266 |

### Table S2. Results from Model II: bivariate with random effects of offspring and mother. Complete fixed effect results from bivariate Model II, using repeated measures of flight initiation distance (FID) on each subadult and adult female individual eastern grey kangaroo. The ‘base’ levels for categorical variables were: *sex* male, *presence of mother/ offspring* none, *year* 2017, and *observer* Observer 1. Bold indicates *p*MCMC (Markov Chain Monte Carlo) < 0.05. Iterations = 750001:3247501. Thinning interval = 2500. Sample size = 1000. DIC: 6973.33

|  | Subadult FID | | Maternal FID | |
| --- | --- | --- | --- | --- |
| Fixed effects | Estimate | *p-MCMC* | Estimate | *p-MCMC* |
| Intercept | **-16.9 (-28.2, -5.8)** | **0.014** | -3.8 (-10.9, 3.7) | 0.316 |
| Age at Observation (in months) | -0.4 (-1.5, 0.8) | 0.520 | - | - |
| Sex (Female) | 0.4 (-0.7, 1.3) | 0.480 | - | - |
| East-west coordinate (in meters) | **0.003 (0.002, 0.005)** | **<0.001** | **0.001 (0.000, 0.002)** | **0.006** |
| Group Size | **-0.3 (-0.7, 0.0)** | **0.050** | **-0.2 (-0.5, 0.0)** | **0.046** |
| Presence of mother/ offspring:  - 1 year-old with mother | -0.2 (-1.1, 0.5) | 0.594 | - | - |
| - mother with Small Pouch Young (<3 months old) | - | - | -0.5 (-1.2, 0.2) | 0.154 |
| - mother with Medium Pouch Young (4-6 months old) | - | - | 0.8 (-0.1, 1.7) | 0.082 |
| - mother with Large Pouch Young (7+ months old) | - | - | **1.3 (0.7, 2.0)** | **<0.001** |
| - mother with 1-year-old offspring | - | - | 0.7 (0.0, 1.5) | 0.086 |
| Test Number | **-0.4 (-0.6, -0.1)** | **0.006** | -0.2 (-0.3, 0.0) | 0.080 |
| Difference in Days from the Previous Test | **0.002 (0.001, 0.004)** | **0.002** | 0.001 (0.000, 0.003) | 0.120 |
| Year  -2018 | **1.8 (0.8, 2.7)** | **<0.001** | 0.2 (-0.5, 0.9) | 0.612 |
| -2019 | **2.5 (1.1, 3.8)** | **<0.001** | 1.1 (-0.4, 2.4) | 0.126 |
| Observer (Observer 2) | - | - | 0.7 (-0.4, 1.8) | 0.226 |

###

### Table S3. Results from the supplementary test on adoptive young Flight Initiation Distance (FID): linear regressions of maternal (biological or adoptive) FID effects on adopted subadult FID in eastern grey kangaroo (n = 6).

|  | Biological Mother | | | Adoptive Mother | | |
| --- | --- | --- | --- | --- | --- | --- |
|  | Estimate | Std Error | Pr(>\|t\|) | Estimate | Std Error | Pr(>\|t\|) |
| Intercept | 7.1 | 5.324, 1.338 | 0.252 | 2.4 | 6.881, 0.345 | 0.753 |
| Maternal FID | 0.4 | 0.634, 0.588 | 0.588 | 0.6 | 0.694, 0.910 | 0.430 |

### MCMC glmm Codes for Model I

We provide a quick run through of the codes here, explaining the different steps. We also share our actual repository on GitHub, including R scripts and the data: <https://github.com/weli-sci/Multivariate-Beh-Surv-Analysis.git>. Data will be archived to Dryad upon acceptance.

**# Data preparation tips**

- Make sure R reads your factors as characters (i.e., special attention to Animal ID or Year whose levels tend to be numbers, and R automatically reads it as numeric variables)
- Before merging files, make sure to remove NAs from the variables – also, a good practice is to have a variable called ‘Data File’ before merging, so after the different files are merged, it is easier to do data cleaning if required
- After merging the files, variables that are not common across different responses will receive NAs – transform those NAs to ‘0’, and we will set the models in the way those 0 values will not be used. ATTENTION! Do this only for explanatory variables.

**# Set a prior####**

# In this case, we have four response variables. We want covariances across all variables for the random effect G1, but residual covariance only for the three last responses listed in the model. We are also fixing the residual variance for the last variable listed to 1 (Juvenile Survival, as it is binary)

library(MCMCglmm)

PriorModel1 <- list(G = list(G1 = list(V = diag(4), nu = 4)),

R = list(R1=list(V=diag(1), nu =1),

R2 = list(V = diag(3), nu = 3,fix=3)))

**# Fitting the model####**

times<-10000 # start with lower values here, and increase depending on autocorrelation

Model_I<-MCMCglmm(cbind(FIDAdFem,AvFIDJuv,AvPYMov, JuvSurv) ~ trait - 1 +

at.level(x=trait,level=1):(East+ GroupSize+ TestNumber+ RelFemAlong+ Observer+ DifferenceDays)+

at.level(x=trait,level=1:2):(Year)+

at.level(x=trait,level=2):(Sex)+

at.level(x=trait,level=3):(AgeCapture+Sex+Year)+

at.level(x=trait,level=4):(Sex+Year),

random = ~ us(trait):MotherID,

rcov = ~idh(at.level(x=trait, level=1)):units+us(at.level(x=trait, level=2:4)):units, family = c("gaussian","gaussian", "gaussian","categorical"),

data = data.merged, prior = PriorModel1, verbose = T,

nitt=1300* times,thin =1* times, burnin =300* times)

plot(Model_I $VCV)

autocorr(Model_I$Sol)

autocorr(Model_I$VCV) # columns that indicate autocorrelation have first lag as ‘1’. columns that indicate autocorrelation have first lag as ‘1’. We want the all values across the consecutive lags to be below 0. Look which lag is <0.1, and use the lag value as the ‘times’ object – that way, we will multiply nitt, thin, and burnin for that value.

summary(Model_I)

save(Model_I,file="Model_I.Rdata")

**# Maternal Repeatability PY Mov#### (Gaussian trait)**

# This is an example of calculating repeatability on pouch young movement – a Gaussian trait

summary(Model_I)

PYMAposterior.repeatability_M <- Model_I$VCV[, "traitAvPYMov:traitAvPYMov.MotherID"]/

(Model_I$VCV[,"traitAvPYMov:traitAvPYMov.MotherID"] +

Model_I$VCV[, "at.level(x = trait, level = 2:4)2:at.level(x = trait, level = 2:4)2.units"])

hist(PYMAposterior.repeatability_M)

posterior.mode(PYMAposterior.repeatability_M)

HPDinterval(PYMAposterior.repeatability_M)

save(PYMAposterior.repeatability_M,file="PYMAposterior.repeatability_M_RepeatabilitiesMCMC_PYM_MotherID.Rdata")

# Within-mother Offspring Repeatability PYM####

summary(Model_I)

PYMAposterior.repeatability_O <- Model_I$VCV[, "at.level(x = trait, level = 2:4)2:at.level(x = trait, level = 2:4)2.units"]/

(Model_I$VCV[,"traitAvPYMov:traitAvPYMov.MotherID"] +

Model_I$VCV[, "at.level(x = trait, level = 2:4)2:at.level(x = trait, level = 2:4)2.units"])

hist(PYMAposterior.repeatability_O)

posterior.mode(PYMAposterior.repeatability_O)

HPDinterval(PYMAposterior.repeatability_O)

save(PYMAposterior.repeatability_O,file=" PYMAposterior.repeatability_O_RepeatabilitiesMCMC.Rdata")

**# Maternal Repeatability Juv Surv#### (Binary trait)**

# This is an example of calculating repeatability on juvenile survival – a non-Gaussian trait

# Data scale back-transformation to original data (0/1).

# Example here on maternal repeatability estimation

library(QGglmm)

RepsSurv_M2<-vector(length=1000)

for (i in 1:1000) {

prevalues <- predict.MCMCglmm(object = Model_I, it=i, type = "terms")

qgrSurv_M2<-QGicc(predict =prevalues,

var.comp = Model_I$VCV[i,"traitJuvSurv.1:traitJuvSurv.1.MotherID"],

var.p = Model_I$VCV[i,"traitJuvSurv.1:traitJuvSurv.1.MotherID"]+

Model_I$VCV[i,"at.level(x = trait, level = 2:4)3:at.level(x = trait, level = 2:4)3.units"],

model = "binom1.logit")

RepsSurv_M2[i]<-qgrSurv_M2$icc.obs

}

save(RepsSurv_M2,file="RepsSurv_M2_RepeatabilitiesMCMC_YoungSurvival_MotherID.Rdata")

plot(as.mcmc(RepsSurv_M2))

posterior.mode(as.mcmc(RepsSurv_M2), adjust = 1)

mean(RepsSurv_M2)

HPDinterval(as.mcmc(RepsSurv_M2))

# Proportions on the data scale

JuvSurvposterior.repeatability_M <- Model_I$VCV[, "traitJuvSurv.1:traitJuvSurv.1.MotherID"]/

(Model_I$VCV[,"traitJuvSurv.1:traitJuvSurv.1.MotherID"] +

Model_I$VCV[, "at.level(x = trait, level = 2:4)3:at.level(x = trait, level = 2:4)3.units"])

hist(JuvSurvposterior.repeatability_M)

posterior.mode(JuvSurvposterior.repeatability_M2)

mean(JuvSurvposterior.repeatability_M)

HPDinterval(JuvSurvposterior.repeatability_M)

### MCMC glmm Codes for Model II

**# Fitting the model####**

library(MCMCglmm)

PriorModelII <- list(G = list(G1 = list(V = diag(2), nu = 2),

G2 = list(V=diag(1), nu =1)),

R = list(V = diag(2), nu = 2))

times<-10000

Model_II<-MCMCglmm(cbind(FIDAdFem,FIDJuv) ~ trait - 1 +

at.level(x=trait,level=1:2):(East+ GroupSize+ TestNumber+ RelFemAlong+ Year+ DifferenceDays)+

at.level(x=trait,level=1):(Observer)+

at.level(x=trait,level=2):(AgeatObs+ Sex),

random = ~ us(trait):MotherID+idh(at.level(x=trait, level=2)):YoungID,

rcov = ~idh(trait):units, family = c("gaussian","gaussian"),

data = data.merged2, prior = PriorModelII, verbose = T,

nitt=1300*times,thin =1*times,burnin =300*times)

plot(Model_II$VCV)

autocorr(Model_II$Sol)

autocorr(Model_II$VCV)

save(Model_II,file="Model_II_CovarianceForQuestion31000It.rdata")

**# Getting repeatability proportions####**

# Maternal-level repeatability proportion

rJuvProportion_M<-((Model_II3$VCV[, "traitFIDJuv:traitFIDJuv.MotherID"])/

((Model_II3$VCV[,"traitFIDJuv:traitFIDJuv.MotherID"])+(Model_II3$VCV[,"at.level(x = trait, level = 2).YoungID"])))

mean(rJuvProportion_M)

HPDinterval(rJuvProportion_M)

# Individual-level repeatability proportion

rJuvProportion_O<-((Model_II$VCV[, "at.level(x = trait, level = 2).YoungID"])/

((Model_II$VCV[,"traitFIDJuv:traitFIDJuv.MotherID"])+(Model_II$VCV[,"at.level(x = trait, level = 2).YoungID"])))

mean(rJuvProportion_O)

HPDinterval(rJuvProportion_O)

#Total repeatability

rJuvProportion_Total<-((Model_II$VCV[, "at.level(x = trait, level = 2).YoungID"])+(Model_II$VCV[,"traitFIDJuv:traitFIDJuv.MotherID"]))/

((Model_II$VCV[,"traitFIDJuv:traitFIDJuv.MotherID"])+(Model_II$VCV[,"at.level(x = trait, level = 2).YoungID"])+

(Model_II$VCV[,"traitFIDAdFem.units"])+

(Model_II$VCV[,"traitFIDJuv.units"]))

mean(rJuvProportion_Total)

HPDinterval(rJuvProportion_Total)

**# Estimating an upper limite heritability####**

h2<-((Model_II$VCV[, "traitFIDAdFem:traitFIDJuv.MotherID"])/

(Model_II$VCV[,"traitFIDAdFem:traitFIDAdFem.MotherID"]))*2

mean(h2)

HPDinterval(h2)
